# Aeration Increases Cadmium (Cd) Retention by Enhancing Iron Plaque Formation and Regulating Pectin Synthesis in the Roots of Rice (*Oryza sativa*) Seedlings

**DOI:** 10.1186/s12284-019-0291-0

**Published:** 2019-05-02

**Authors:** Hubo Li, Xiuwen Zheng, Longxing Tao, Yongjie Yang, Lei Gao, Jie Xiong

**Affiliations:** 1 School of Life Sciences and Medicine, Zhejiang Sci-Tech University, Hangzhou, 310018 People’s Republic of China; 2 State Key Laboratory of Rice Biology, China National Rice Research Institute, Hangzhou, 310006 People’s Republic of China

**Keywords:** Aeration, Cadmium, Cell wall, Pectin, Iron plaque, Oxygen, Rice (*Oryza sativa*)

## Abstract

**Background:**

Aeration and water management increasing rhizosphere oxygen amount significantly promote rice (*Oryza sativa*) growth and yield, but the effect of root aeration on cadmium (Cd) toxicity and accumulation in rice seedlings under hydroponic culture remains unclear.

**Results:**

Results showed that aeration promoted rice seedling growth and alleviated Cd toxicity. Transverse section discovered that Cd accelerated root mature and senescence while aeration delayed the mature and senescence of roots. Non-invasive Micro-test Technology (NMT) showed that aeration increased net O_2_ and Cd^2+^ influxes on the surface of roots while decreased net Cd^2+^ influx in xylem. Perls blue staining showed that aeration and Cd treatments increased iron plaque formation on the surface of roots. Results of metal concentration analysis showed that besides increasing Cd retention in iron plaque, aeration also increasing Cd retention in the cell wall of rice roots. Cell wall component analysis showed that aeration not only increased pectin content but also decreased pectin methylesterification degree (PMD) by increasing pectin methylesterase (PME) activity.

**Conclusions:**

All of these results indicate that aeration not only delays root mature and senescence but also increases Cd retention in roots by enhancing iron plaque formation and regulating pectin synthesis in the roots of rice seedlings.

**Electronic supplementary material:**

The online version of this article (10.1186/s12284-019-0291-0) contains supplementary material, which is available to authorized users.

## Background

Cadmium (Cd) is a toxic heavy metal to rice (*Oryza sativa*) and mainly ionized as Cd^2+^ in the rhizosphere (Yoneyama et al. 2015). As a highly mobile and soluble metal, Cd exposure reduces crops yield and does harm to humans’ health even at low concentrations. Rice is a staple food consumed by half the world’s population, especially in East and South Asia, the Middle East, Latin America, and the West Indies (Sharif et al. 2014). Due to daily consumption, Cd in rice grains poses a latent health problem to humans through food chains and leads to chronic toxicity. The outbreak of “Itai-itai disease” in the mid-twentieth century in Japan is due to consumption of Cd-contaminated rice (Horiguchi et al. 1994). Even in recent years, Cd exposure in general Japanese population can be as high as 3–4 mg kg^−1^ body weights every week (Tsukahara et al. 2003). Furthermore, as an export commodity, rice also has posed an increasing threat to human health globally due to the contamination by Cd. Therefore, it is necessary to reduce the concentration of rice Cd below the allowable level indicated by the Codex Alimentarius Commission of FAO/WHO (CODEX 2006).

In response to Cd toxicity, plants have evolved several protective mechanisms against Cd toxicity, including avoidance and tolerance strategies (Dalcorso et al. 2010). Plants can prevent Cd from entering into plant cells, which is referred to as an avoidance strategy. To provide a barrier for metals toxic, formation of iron plaque on root surface has been considered as a crucial survival strategy for wetland plants, such as rice, in anoxic and flooded environments (Smolders and Roelofs 1996; Chabbi 1999). The formation of iron plaque on the root surface due to the radial oxygen loss (ROL) and oxidants in the rhizosphere, where ferrous is subsequently oxidized to ferric iron with the precipitation of iron oxide or hydroxide on the root surface (Chen et al. 1980; Taylor et al. 1984). Root iron plaque has been shown to impede the entry of metal ions, such as Cd, Al, Pb and Zn in plants (Chen et al. 2006; Liu et al. 2007; Deng et al. 2009). On the other hand, plant cell wall is another barrier to prevent Cd from entering and damaging the protoplast. Both the primary and secondary cell wall have an array of defensive mechanisms that can be adapted to cope with Cd (Lux et al. 2011). The main components of plant cell walls are cellulose, hemicellulose, pectin, and glycoprotein. More than 25 years ago, Poschenrieder et al. (1989) presumed that Cd increases the cross linking of pectin in the middle lamella and inhibits cell expansion growth. Now it has been well established that negatively charged pectin strongly binds positively charged cations to form a stable gel with each other in cell walls (Kohn 1987; Liners et al. 1989), and there is a close positive correlation between pectin content and heavy metal accumulation in cell walls (Schmohl and Horst 2000; Paynel et al. 2009; Xiong et al. 2009b). It is now well suggested that pectin is polymerized in the cis Golgi, methylesterified in the medial Golgi and substituted with side chains in the trans Golgi cisternae (Micheli 2001). Pectin is secreted in a highly methylesterified form and demethylesterified by pectin methylesterase (PME), pectin methylesterification degree (PMD) determines negative charges and has a close negative correlation with heavy metal adsorption in the cell walls of plant roots (Eticha et al. 2005; Yang et al. 2008). Ishikawa et al. (2011) suggested Cd uptake could be mediated by transporters. In fact, Cd entrance into root cells via transporter *OsNRAMP5* or *OsIRT1* have been proved and *OsNRAMP5* is predominantly applied (Nakanishi et al. 2006; Sasaki et al. 2012). In addition, higher expression of *OsNRAMP1* in root could enhance Cd accumulation in shoot of rice, indicating that *OsNRAMP1* was also related with Cd uptake and transport (Takahashi et al. 2011). The abilities of resistance to Cd stress inside the cells are referred to as tolerance strategy (Choppala et al. 2014). In rice, phytochelatins (PC) acting as Cd chelator plays a key role in Cd detoxification (Yadav et al. 2010). PC chelates Cd in the cytosol and forms complexes with Cd. Then the Cd-PCs complexes are sequestered in the vacuoles via specific transporters located at tonoplast (Ueno et al. 2010; Miyadate et al. 2011).

Several approaches have been proposed to reduce Cd concentration in rice culture, such as soil dressing, chemical remediation, and breeding or engineering low-Cd-accumulating cultivars (Ishikawa et al. 2006; Uraguchi et al. 2006; Makino et al. 2008; Ueno et al. 2011). As a commonly used type of water management, flooding treatment often leads to a reduction in oxygen or even oxygen deficiency due to the slower diffusion of gases in water than in air and exacerbated by the competition for oxygen consumption by soil microorganisms (Bailey-Serres and Voesenek 2008). Numerous studies have revealed that root uptake of ions and transport of ions to shoots is severely inhibited under waterlogging (anoxia) conditions (Pang et al. 2004; Smethurst et al. 2005). It is reported that Cd is mainly present as free Cd^2+^ in soil under aerobic condition regardless of soil redox potential (Du Laing et al. 2009). It was suggested that Cd in soil existed in insoluble form as sulfide in the flooding period, flooding during the growing season, especially during later stages of plant growth, can effectively reduce Cd concentrations in rice grains. So continuous flooding treatment is considered to be a simple method to inhibit Cd absorption in rice culture by reducing oxygen and free Cd^2+^ concentration (Daum et al. 2001; Cattani et al. 2008). Hu et al. (2013a, 2013b) reported that the HCl-extractable Cd concentrations decreased significantly with increasing irrigation from aerobic to flooded conditions. In contrast, Yang et al. (2009) reported that compared to the well-watered treatment, moderate soil drying (MD, re-watered when soil water potential decreased to − 20 kPa) and severe soil drying (SD, re-watered when soil water potential decreased to − 40 kPa) increased Cd content in roots while they reduced it in the straw. MD reduced Cd content by 19–21% in the grain and by 40% in milled rice. The SD significantly increased Cd content in the grain but reduced it in milled rice (Yang et al. 2009). Furthermore, Yang et al. (2017) also reported that moderate wetting and drying increases rice yield and reduce water use, grain arsenic level and methane emission.

However, soil drying treatment is quite different from root aeration treatment. Although soil drying can increase rhizosphere oxygen amount, it also can seriously affect rice growth and development. Oxygen is positive for the growth of rice, root aeration and other methods to increase the rhizosphere oxygen amount significantly promoted rice growth and yield in the paddy field (Zhu et al. 2015; Yang et al. 2017). But to date, the effect and mechanism of root aeration on Cd accumulation in rice seedlings remains unclear. In this study, in order to investigate the effect of root aeration on Cd accumulation in rice seedlings, hydroponic culture was used to avoid interferes of different forms of Cd caused by soil redox potential and pH value in soil.

## Methods

### Rice Hydroponic Culture, Cd and Aeration Treatment

Healthy rice (*O. sativa* L., cv. Nipponbare) seeds were surface sterilized using 5% sodium hypochlorite for 15 min, and the seeds were then washed 3 times with sterilized water. After soaking in sterilized water at 37 °C for 48 h, the seeds were germinated in Petri dishes with wet filter paper at 37 °C for another 24 h. Germinated seeds were then transferred to a net tray floating on a container filled with sterilized water (pH 5.2) for 1 week. Then, sterilized water was replaced with improved Yoshida rice nutrient solution in full strength (Yoshida et al. 1976) for another 2 weeks. The Yoshida rice nutrient solution contains the following components (in mM): NH_4_NO_3_, 1.43; CaCl_2_, 1.00; MgSO_4_, 1.64; K_2_SO_4_, 1.00; NaH_2_PO_4_, 0.32; FeCl_3_, 3.6 × 10^− 2^; MnCl_2_, 9.4 × 10^− 3^; H_3_BO_3_, 1.9 × 10^− 2^; (NH_4_)_6_Mo_7_O_24_, 5.17 × 10^− 4^; ZnSO_4_, 1.52 × 10^− 4^; CuSO_4_, 1.36 × 10^− 4^ and Na_2_SiO_3_, 5.00 × 10^− 3^. FeCl_3_ was replaced with 3.1 × 10^− 2^ mM Fe^2+^-EDTA as source of Fe. Finally, 3-week-old uniform seedlings were transplanted into 5-L black plastic pot containing full strength Yoshida rice nutrient solution (4 seedlings per pot), with either no Cd (control) or 50 μM Cd (CdCl_2_). Half the rice seedlings were aerated using an air pump (30 min per hour, O_2_ concentration in nutrient solution was around 7.0 ± 0.5 mg/L), while the other half were continuously treated as previously described. Totally, there were 4 treatments: control, aeration, 50 μM Cd and aeration plus 50 μM Cd. Each treatment was set up in triplicate to ensure the reproducibility of the results. The pH of the nutrient solution was adjusted to 5.2 with NaOH or HCl every 3 d, and the nutrient solution was refreshed every 6 d. All seedlings were grown in a plant growth chamber with 14 h at 30 °C: 10 h at 24 °C day: night photo- and thermoperiod, and 80% relative humidity.

### Growth Analysis

Root length and shoot height were measured with an accuracy of ±1 mm with the help of a ruler. For biomass determination, the shoot and root of seedlings were separated and dried at 105 °C for 1 h and then dried at 80 °C for another 48 h. After cooling, the dry weight of shoot and root were weighed and recorded. For root diameter determination, roots were hand sectioned and transverse sections were taken from 0.5 cm to 1 cm behind the root apex; the root diameter was then measured and analyzed by microscope and software (DM4000B, Leica, Wetzlar, Germany). At least 5 repeats were measured for each treatment.

### Determination of Net Cd^2+^ and O_2_ Fluxes

Net O_2_ and Cd^2+^ flux were measured in YoungerUSA Xuyue (Beijing) BioFunction Institute by using Non-invasive Micro-test Technology (NMT) (NMT100 Series, YoungerUSA LLC, Amherst, MA, USA; Xuyue (Beijing) Sci. & Tech. Co., Ltd., Beijing, China and imFluxes V2.0 (YoungerUSA LLC, Amherst, MA, USA) Software, which is capable of integrating and coordinating differential voltage signal collections, motion control, and image capture simultaneously. For net Cd^2+^ flux determination, a 1-cm length of filling liquid (10 mM Cd (NO_3_)_2_, 0.1 mM KCl) was injected into a pre-pulled and salinized glass microsensor (Φ4.5 ± 0.5 μm, XY-CGQ-01, YoungerUSA), and then the glass microsensor was front filled with 40–50 μm column of Cd^2+^ liquid exchange reagent (LIX, YoungerUSA). Finally, an Ag/AgCl wire microsensor holder YG003-Y11, (YoungerUSA) was inserted in the back of the microsensor to make electrical contact with the electrolyte solution. In net Cd^2+^ flux measurement, standard solutions containing 10 μM or 100 μM CdCl_2_ as well as 0.1 mM KCl, 0.1 mM CaCl_2_ and 0.3 mM MES at pH 5.4 were used to calibrate the microelectrode. Calibration was achieved when the Nernst slope drawn through two points was 29 ± 3 mV/decade. After 2 week treatments, 3 cm length roots from the root apex were cut and rinsed in distilled water, and then transferred to a plastic Petri dish containing a test solution of 10 μM CdCl_2_ at pH 5.4. After 10 min of equilibration, tests were carried out on root micro-zones with a step pitch of 30 μm. In net O_2_ flux measurement, The Pt/Ir polarographic oxygen microsensor (Φ20 ± 5 μm, XY-CGQ-501, YoungerUSA) were used for detecting dissolved oxygen in –750 mV polarization voltage. The oxygen microsensor was calibrated with nitrogen-saturated and control cultural solution. The different root zones were distinguished by their morphological properties as observed under a microscope. Measurements made on the root surface 200 and 500 μm from the root apex represented the meristematic zone and the growth zone respectively. Transverse section at 500 μm from the root apex were taken to measure fluxes in xylem. Steady-state Cd^2+^ and O_2_ flux were recorded for 10 min, and each measurement was repeated three times. The micro-volt differences were then exported as raw data before they were imported and converted into net fluxes by using the JCal V3.3 (a free MS Excel spreadsheet, youngerusa.com or xbi.org).

### Analysis of Iron Plaque

Perls blue staining of iron plaque was performed according to the method of Yokosho et al. (2009). Briefly, equal amounts of solutions of 4% (*v*/*v*) HCl and 4% (*w*/*v*) potassium ferrocyanide were mixed immediately prior to use. Roots of rice seedlings (3-week-old) were exposed to the staining solution and vacuum infiltrated for 15 min. The staining was observed under stereomicroscope.

Iron plaque was extracted from fresh root surface using the dithionite-citrate-bicarbonate method (DCB) (Taylor and Crowder 1983). Fresh root material was placed in a solution of 40 ml of 0.3 M sodium citrate (Na_3_C_6_H_5_O_7_·2H_2_O) and 5 ml of 1.0 M sodium bicarbonate (NaHCO_3_) at 25 °C. Three grams of sodium dithionite (Na_2_S_2_O_4_) was added and the mixture was agitated continuously for 3 h. After the 3-h extraction period, the wash was collected and the roots were washed three times with 15 ml of deionized water. The resulting solution was made to volume (100 ml) with deionized water. Cd and Fe content in the resulting solution were then measured using an atomic absorption spectrometer (AA-6800, Shimadzu, Kyoto, Japan).

### Analysis of Cd and Fe Content in Rice Tissue

After the DCB extraction, seedlings were dissected into root and shoot. Half the root was used to analyze distribution of Cd in root cells according to the method of Carrier et al. (2003) with tiny modifications. Each 5 g sample was homogenized in 10 ml of chilled extraction buffer containing 50 mM Hepes (pH 7.5), 500 mM sucrose, 1 mM DTT, 5 mM ascorbate and 1% polyvinylpolypyrrolidone (PVPP). The homogenate was centrifuged at 500 *g* for 5 min to isolate the cell wall fraction. Then the supernatant was centrifuged at 20,000 *g* for 45 min to sediment cell organelles, the resultant supernatant solution was referred to as the soluble fraction. All steps were performed at 4 °C. The fractions of the samples were digested in a mixture of HNO_3_ and HClO_4_ (4:1, *v*/*v*) at 120 °C for at least 3 h. Another half of root and shoot were dried at 105 °C for 1 h and then were dried for another 48 h; the dry samples were digested in mixture of 4:1 (*v*/*v*) HNO_3_ and HClO_4_ at 120 °C for at least 3 h. Cd and Fe content also were quantified using an atomic absorption spectrometer (AA-6800, Shimadzu, Kyoto, Japan).

### Cell Wall Fractionation and Measurement

Based on the methods of Zhong and Läuchli (1993), the crude cell walls were prepared and fractionated into four fractions: pectin, hemicellulose1 (HC1), hemicellulose 2 (HC2), and cellulose. Uronic acid content in pectin, HC1 and HC2 was assayed according to the method of Blumenkrantz and Asboe-Hansen (1973). Galacturonic acid (GalA) was used as a calibration standard and pectin content was expressed as GalA equivalents. Cellulose content was assayed according to the methods of Correa-Aragunde et al. (2008).

### PME Activity Assay

PME was extracted according to the method of Ren and Kermode (2000) with modification; fresh roots were fully ground in the PME extraction buffer (0.1 M citrate acid, 0.2 M Na_2_HPO_4_, and 1.0 M NaCl, pH 5.0) at 4 °C. The homogenized slurry was transferred to an Eppendorf tube and incubated on ice for 1 h, during which it was vibrated 3 times at 20 min intervals, and then the tube was centrifuged for 10 min at 15,000 *g* at 4 °C. The supernatant was collected for PME activity determination. PME activity was determined according to the method of Richard et al. (1994). The extract was added to the PME activity assay buffer (0.5% (*w*/*v*) citrus pectin, 0.2 M NaCl, and 0.15% (*w*/*v*) methyl red (pH 6.8), then the mixture was incubated for 1.5 h at 37 °C. Pectin de-esterification decreases the pH, thus changing the color from yellow to red. The color change was recorded at 525 nm with a spectrophotometer. A calibration curve was obtained by adding 10–300 μl 0.01 M HCl to 4 ml PME activity assay buffer and the respective optical density (OD) values were measured at 525 nm.

### Determination of Degree of Pectin Methylesterification

The degree of pectin methylesterification was calculated from the ratio of the methanol and GalA content according to the method of Anthon and Barrett (2008). Methanol was determined using AO and Purpald as described in the method of Anthon and Barrett (Anthon and Barrett 2004), and GalA content was assayed according to the method of Blumenkrantz and Asboe-Hansen (1973).

### Statistical Analysis

The data were analyzed by analysis of variance (ANOVA). ANOVA and the difference (least-significant difference, LSD) test were employed to determine differences among the treatments at *P* < 0.05.

## Results

### Effects of Cd and Aeration on Rice Growth and Root Structure

**Fig. 1:**
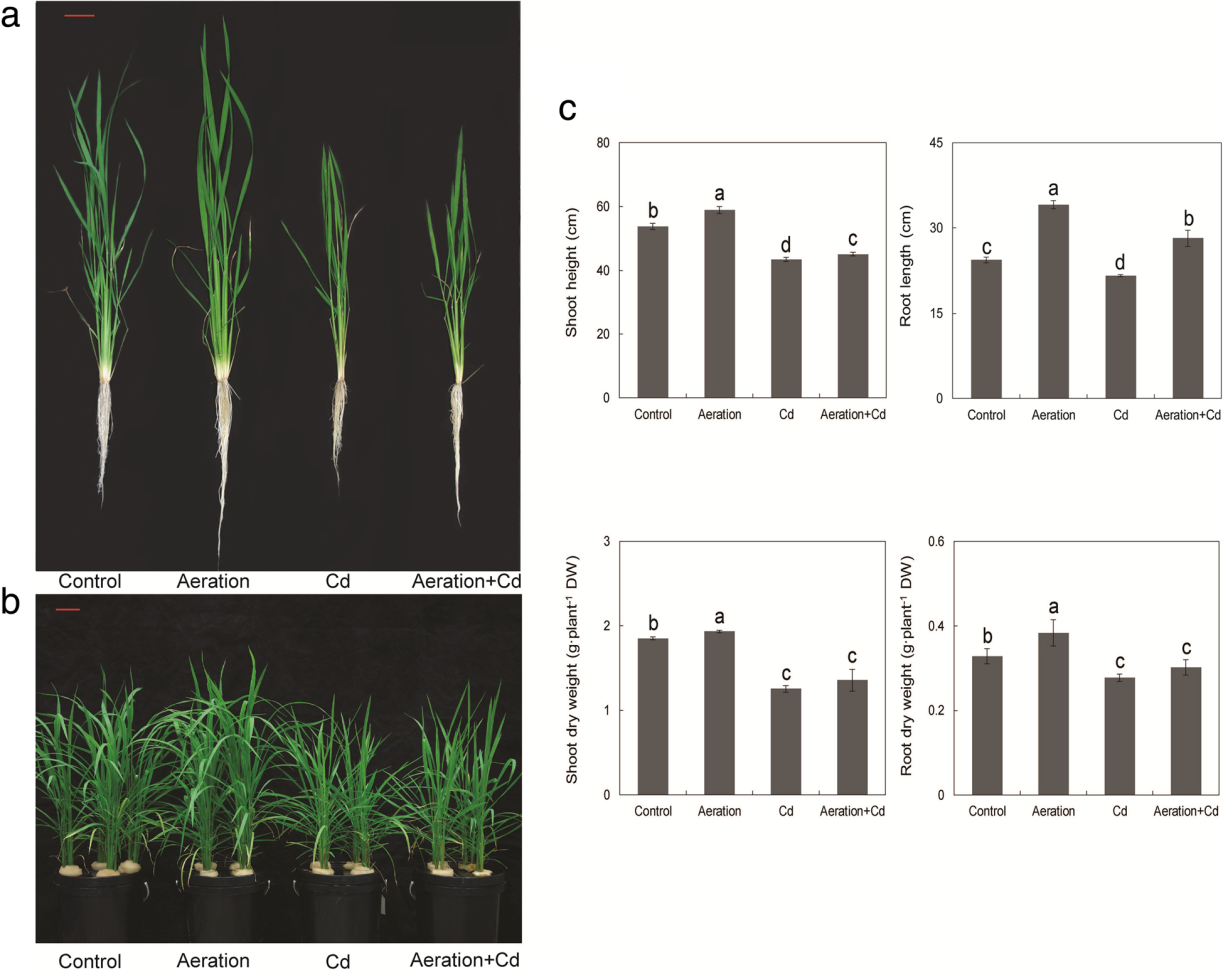
Effects of aeration or/and 50 μM CdCl_2_ treatments on the growth of rice seedling. **a** Intact seedling growth phenotype, bar indicates 5 cm; **b** Hydroponic culture condition, bar indicates 5 cm; **c** Shoot height, root length, shoot dry weight and root dry weight of rice seedlings. The 3-week-old rice seedlings under hydroponic culture were aerated with air pump (30 min per hour) in the absence or presence of 50 μM CdCl_2_ for 14 d. The values are means ± SE (*n* = 8). Different letters on bar indicate significant differences at *P* < 0.05

Compared with control, Cd treatment not only inhibited shoot height and root length but also reduced biomass accumulation (dry weight of shoot and root) of rice seedlings (Fig. 1). In the absence of Cd, aeration showed an obvious growth-promoting effect on rice seedlings, statistical results showed that aeration significantly increased shoot height, root length and dry weight. In the presence of Cd, aeration alleviated Cd-caused inhibitions on shoot height and root length, although it had not significant effect on dry weight of rice seedlings (Fig. 1). Totally, aeration slightly alleviated Cd toxicity to rice seedlings.

**Fig. 2:**
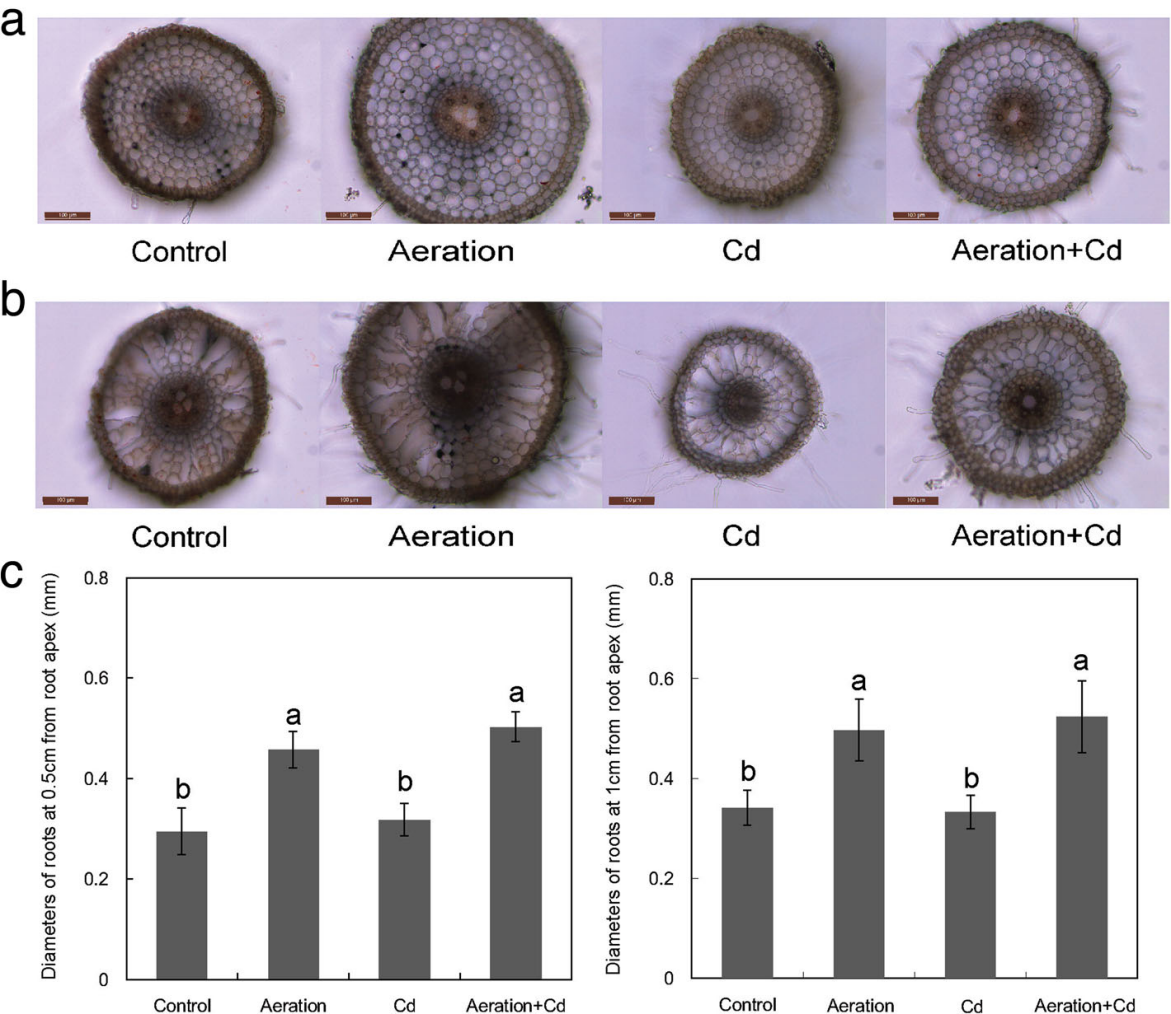
Effects of aeration or/and 50 μM CdCl_2_ treatments on the structure of rice root. **a** Transverse section at 0.5 cm from root apex, bar indicates 100 μm; **b** Transverse sections of root at 1 cm from root apex, bar indicates 100 μm; **c** Diameters of roots at 0.5 cm and 1 cm from root apex. The 3-week-old rice seedlings under hydroponic culture were aerated with air pump (30 min per hour) in the absence or presence of 50 μM CdCl_2_ for 14 d. The values are means ± SE (*n* = 5). Different letters on bar indicate significant differences at *P* < 0.05

Results of transverse section showed that aeration increased the diameter of root by increasing cell size rather than cell layer (Fig. 2a). In addition, transverse section of root at 1 cm from root apex showed that aeration slightly inhibited aerenchyma development (Fig. 2b). In the control, many lacunae extended radially from the endodermis to the outer cortex were separated by narrow sections of parenchyma cells, while aerated root showed more parenchyma cells with less aerenchyma (Fig. 2b). In the presence of Cd, transverse section of root at 1 cm from root apex showed that Cd treatment obviously promoted aerenchyma development, lacunae extended radially from the endodermis to the outer cortex with limited parenchyma cells. Aeration alleviated Cd-induced aerenchyma formation and increased parenchyma cells.

### Effects of Cd and Aeration on net O_2_ and Cd^2+^ Fluxes in Rice Root

**Fig. 3:**
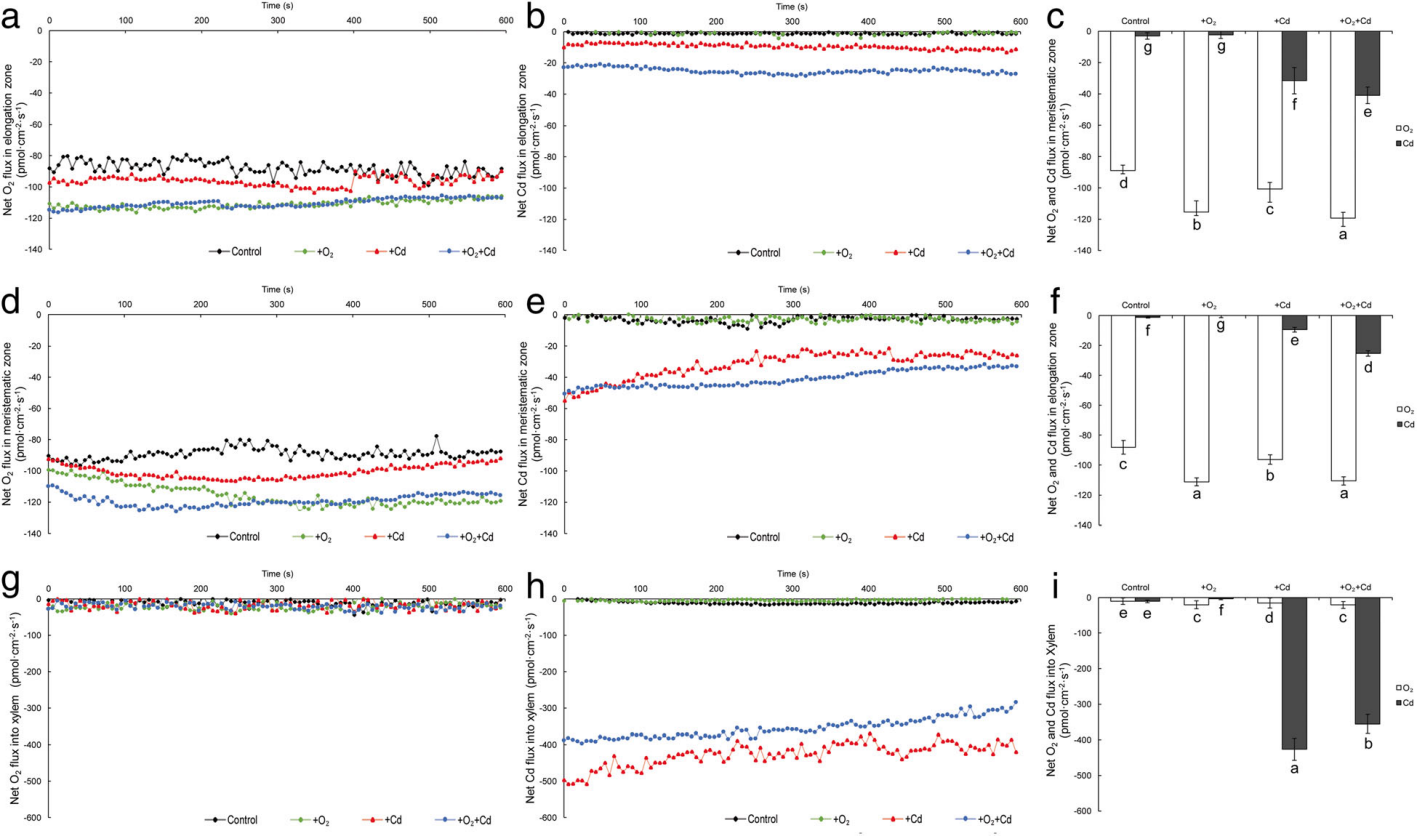
Effects of aeration or/and 50 μM CdCl_2_ treatments on net O_2_ and Cd^2+^ fluxes in the roots of rice seedling. **a** Time-course of Net O_2_ flux on the surface of root at 500 μm from root apex (elongation zone); **b** Time-course of Net Cd^2+^ flux on the surface of root at 500 μm from apex (elongation zone); **c** Statistical results of steady-state net O_2_ and Cd^2+^ fluxes on the surface of root at 500 μm from apex (elongation zone); **d** Time-course of Net O_2_ flux on the surface of root at 200 μm from root apex (meristematic zone); **e** Time-course of Net Cd^2+^ flux on the surface of root at 200 μm from apex (meristematic zone); **f** Statistical results of steady-state net O_2_ and Cd^2+^ fluxes on the surface of root at 200 μm from apex (meristematic zone); **g** Time-course of Net O_2_ flux in the xylem of root at 500 μm from root apex (elongation zone); **h** Time-course of Net Cd^2+^ flux in the xylem of root at 500 μm from apex (elongation zone); **i** Statistical results of steady-state net O_2_ and Cd^2+^ fluxes in the xylem of root at 500 μm from apex (elongation zone). The 3-week-old rice seedlings under hydroponic culture were aerated with air pump (30 min per hour) in the absence or presence of 50 μM CdCl_2_ for 14 d. The values are means ± SE (*n* = 100). Different letters on bar indicate significant differences at *P* < 0.05

Effects of Cd and aeration on net O_2_ and Cd^2+^ fluxes in rice root were investigated using NMT, a positive net flux represented efflux and a negative net flux represented influx. The results showed that obvious net O_2_ fluxes were detected in elongation and meristematic zones rather than xylem (Fig. 3). In contrast, intensive net Cd^2+^ flux was detected in xylem, less net Cd^2+^ flux was detected in meristematic zone and the least net Cd^2+^ flux was detected in elongation zone. In addition, net Cd^2+^ flux in meristematic zone decreased gradually as the duration. Aeration obviously increased net O_2_ fluxes in elongation and meristematic zones of rice root with or without Cd stress. Cd treatment increased net O_2_ fluxes in elongation and meristematic zones of rice root at the first several minutes, and then increased net O_2_ fluxes recovered to the control level gradually. As to net Cd^2+^ flux, aeration obviously increased net Cd^2+^ flux in elongation zone while decreased net Cd^2+^ flux in xylem. Aeration also slightly increased net Cd^2+^ flux in meristematic zone, although net Cd^2+^ flux decreased gradually as the duration.

### Effects of Cd and Aeration Treatments on Iron Plaque

**Fig. 4:**
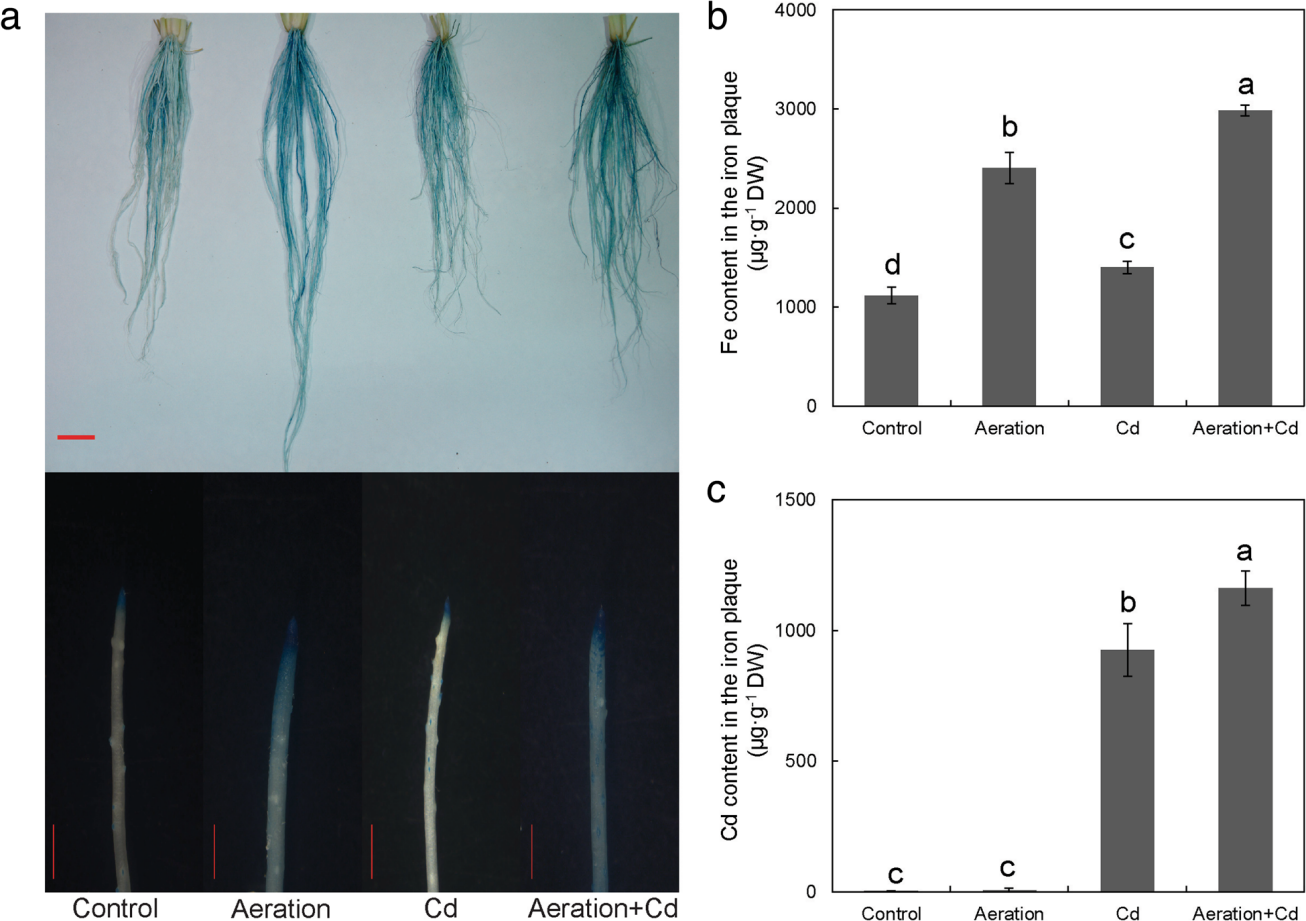
Effects of aeration or/and 50 μM CdCl_2_ treatments on Fe and Cd content in the iron plaque of rice root. **a** Perls blue staining of iron plaque on the surface of rice roots, upper bar indicates 5 cm and lower bar indicate 1 cm; **b** Fe content in the iron plaque of rice roots; **c** Cd content in the iron plaque of rice roots. The 3-week-old rice seedlings under hydroponic culture were aerated with air pump (30 min per hour) in the absence or presence of 50 μM CdCl_2_ for 14 d. The values are means ± SE (*n* = 3). Different letters on bar indicate significant differences at *P* < 0.05

Results of Perls blue staining showed that aeration significantly increase blue color on the surface of root tip in the absence of Cd stress. Meanwhile, Cd treatment also slightly increased blue color on the surface of root tip, Cd plus aeration treatment increased the most obvious blue color on the surface of root tip (Fig. 4a). Metal content analysis showed that aeration significantly increased Fe content in iron plaque of rice roots, regardless of whether it was exposed to Cd treatment or not (Fig. 4b). Furthermore, Cd treatment also had a certain effect on promoting iron plaque formation (Fig. 4b). In addition, Cd also was detected in the iron plaque and aeration increased Cd content in the iron plaque of rice roots under Cd treatment (Fig. 4c).

### Effects of Cd and Aeration Treatments on Cd and Fe Contents in Rice Seedlings

**Fig. 5:**
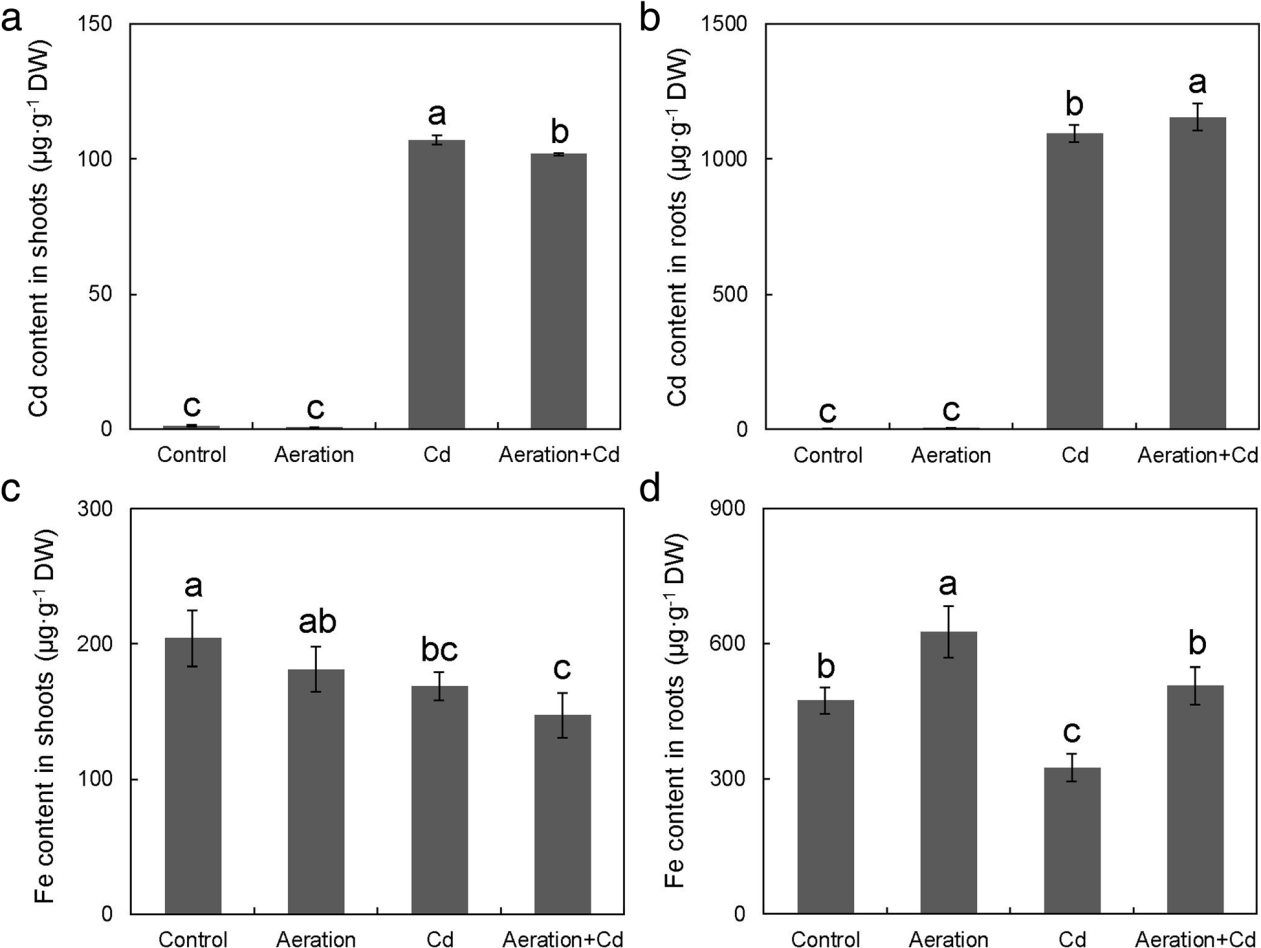
Effects of aeration or/and 50 μM CdCl_2_ treatments on Cd and Fe content in shoots and roots of rice seedling. **a** Cd content in shoots; **b** Cd content in roots; **c** Fe content in shoots; **d** Fe content in roots. The 3-week-old rice seedlings under hydroponic culture were aerated with air pump (30 min per hour) in the absence or presence of 50 μM CdCl_2_ for 14 d. The values are means ± SE (*n* = 3). Different letters on bar indicate significant differences at *P* < 0.05

The results showed that 50 μM CdCl_2_ treatment significantly increased Cd content in both shoots and roots (after the DCB extraction of iron plaque) of rice seedlings (Fig. 5a, b). However, aeration increased Cd content in roots (after the DCB extraction of iron plaque) but decreased Cd content in shoots. As to Fe content in rice seedlings without Cd treatment (Fig. 5c, d), aeration increased Fe content in roots but had no obvious effect on Fe content in shoots. Meanwhile, Cd treatment decreased Fe contents in both shoots and roots (after the DCB extraction of iron plaque) of rice seedlings, aeration increased Fe content in roots (after the DCB extraction of iron plaque) but had no obvious effect on Fe content in shoots.

**Fig. 6:**
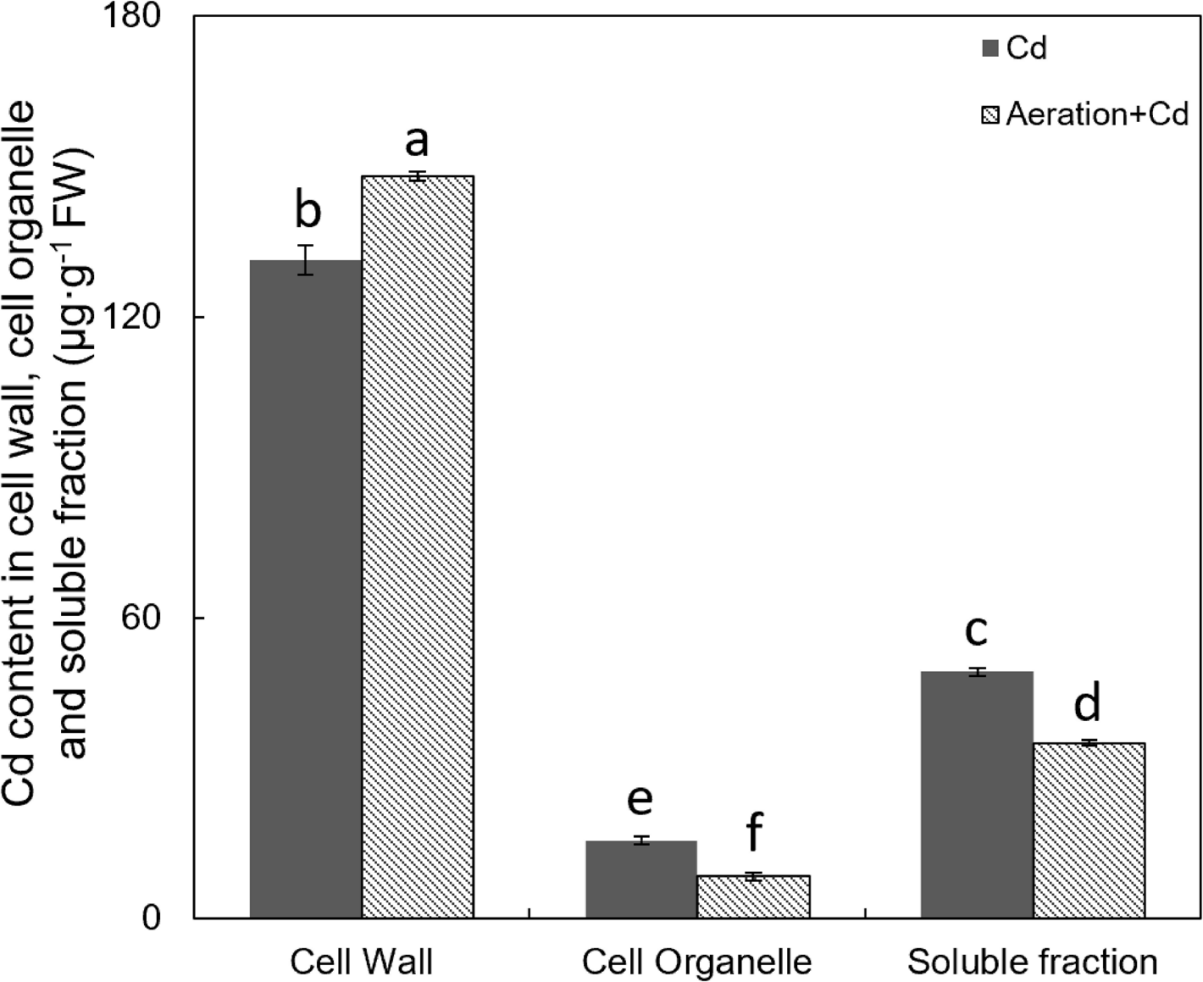
Effects of aeration or/and 50 μM CdCl_2_ treatments on the location of Cd in rice root cells. Figure indicates Cd content in cell wall, cell organelle and soluble fraction. The 3-week-old rice seedlings under hydroponic culture were aerated with air pump (30 min per hour) in the absence or presence of 50 μM CdCl_2_ for 14 d. The values are means ± SE (*n* = 3). Different letters on bar indicate significant differences at *P* < 0.05

Distribution analysis of Cd in rice root cells (after the DCB extraction of iron plaque) showed most of Cd accumulated in cell walls and the least amount of Cd in cell organelles. Aeration increased Cd content in cell walls but decreased Cd contents in cell organelles and soluble fractions (Fig. 6).

### Effects of Cd and Aeration Treatments on Cell Wall Components in Rice Roots

**Fig. 7:**
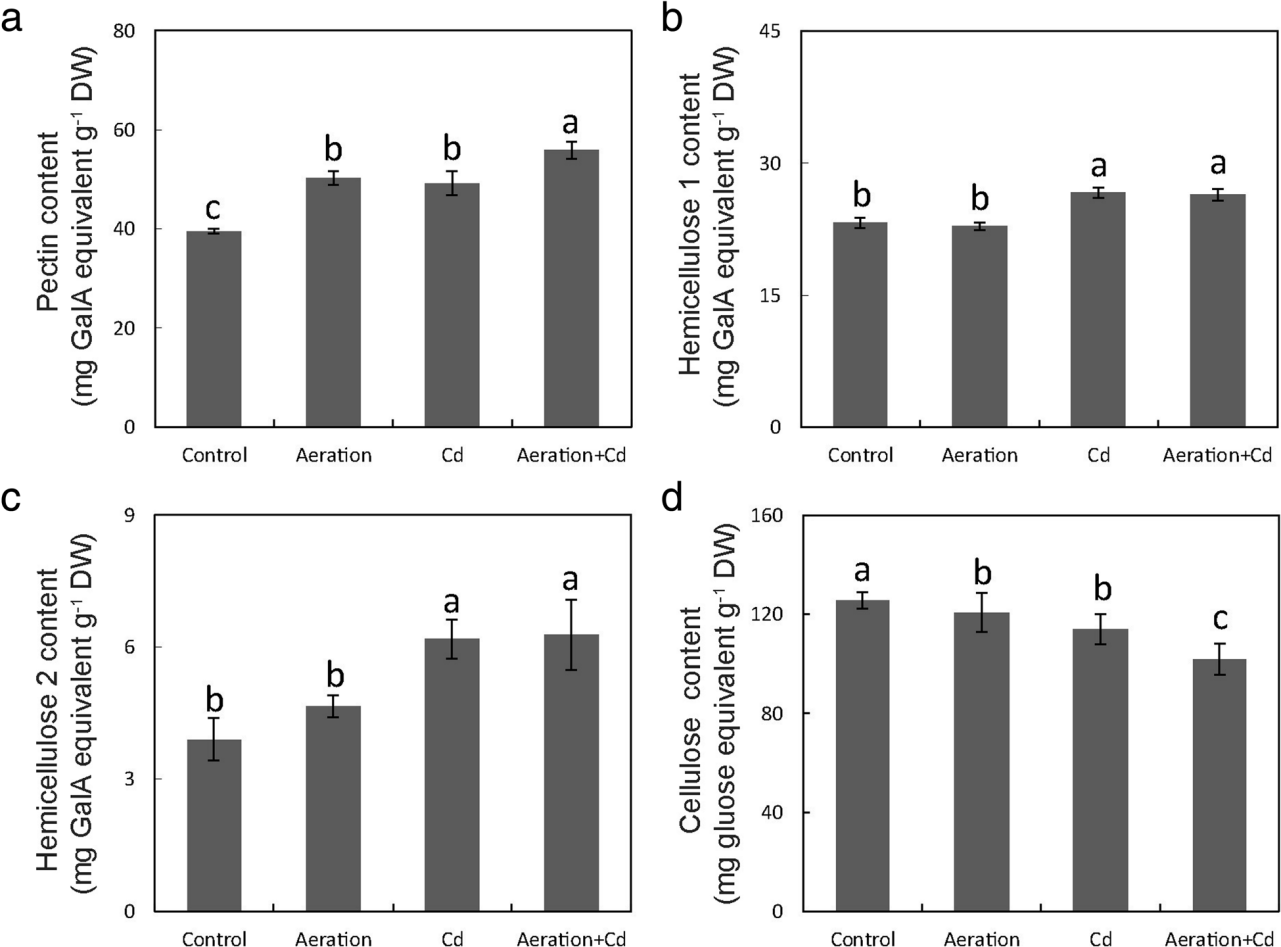
Effects of aeration or/and 50 μM CdCl_2_ treatments on cell wall components in rice root. **a** Pectin content in the cell wall of root; **b** Hemicellulose 1 (HC1) content in the cell wall of root; **c** Hemicellulose 2 (HC2) content in the cell wall of root; **d** Cellulose content in the cell wall of root. The 3-week-old rice seedlings under hydroponic culture were aerated with air pump (30 min per hour) in the absence or presence of 50 μM CdCl_2_ for 14 d. The values are means ± SE (*n* = 3). Different letters on bar indicate significant differences at *P* < 0.05

Cell wall components were divided into pectin, HC1, HC2 and cellulose with graduation extraction method. Compared with control, aeration treatment increased pectin content but decreased cellulose content in the cell wall of rice roots (Fig. 7). Cd treatment increased pectin, HC1 and HC2 contents but decreased cellulose content. Cd plus aeration treatment induced the most obvious increases of pectin, HC1 and HC2 contents in the cell wall of rice roots, although it decreased cell wall cellulose content obviously.

### Effects of Cd and Aeration Treatments on Pectin Methylesterification and PME Activity

**Fig. 8:**
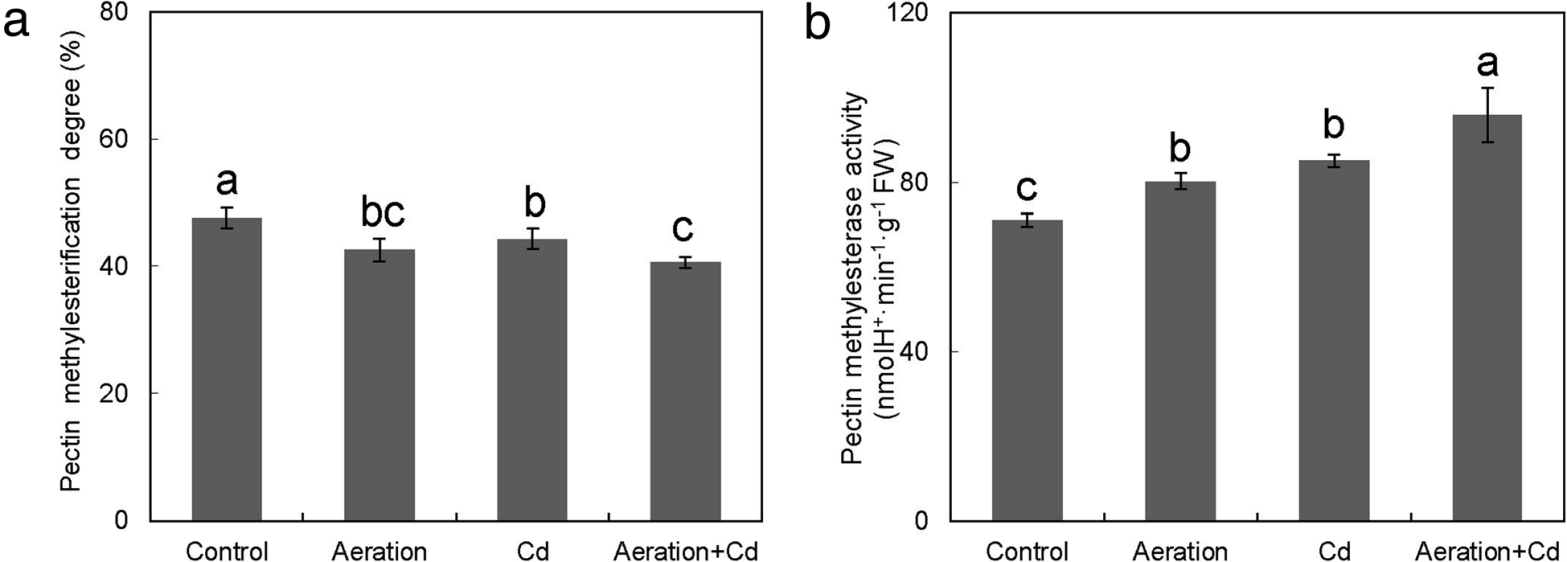
Effects of aeration or/and 50 μM CdCl_2_ treatments on pectin synthesis in the cell wall of rice root. **a** Pectin methylesterification degree (PMD) in rice root; **b** Pectin methylesterase (PME) activity in rice root. The 3-week-old rice seedlings under hydroponic culture were aerated with air pump (30 min per hour) in the absence or presence of 50 μM CdCl_2_ for 14 d. The values are means ± SE (*n* = 3). Different letters on bar indicate significant differences at *P* < 0.05

Compared with control, Cd treatment decreased pectin methylesterification degree and increased PME activity in the cell wall of rice roots (Fig. 8). Aeration treatment also decreased pectin methylesterification degree and increased PME activity in the cell wall of rice roots. Cd plus aeration treatment has the most intensive effects on decreasing pectin methylesterification degree and increasing PME activity in the cell wall of rice roots.

## Discussion

### Aeration Delays Cd-accelerated Mature and Senescence of Rice Roots

In the absence of Cd, aeration significantly increased root length (Fig. 1), indirectly proves aeration delays root mature and senescence. In the presence of Cd, aeration alleviated Cd-caused inhibitions on and root length rather than dry weight (Fig. 1), indicating root elongation duration rather than root amount is regulated by aeration. In addition, aeration increased Cd content in roots (Fig. 5c) indicates aeration alleviates Cd toxicity by increasing Cd tolerance rather than by decreasing Cd absorption in rice roots. Figure 6 shows Cd in rice root was mainly restricted in the cell wall; it is a possible reason for the decreased Cd content in rice shoots in Fig. 5a.

Cd stress produces a series of toxic phenomenon in rice roots, and inhibition of root growth is one of the most significant symptoms (Xiong et al. 2009a). In this study, Cd significantly inhibited root length and dry weight (Fig. 1). Under disadvantage growth conditions, roots also undergo structural and functional modifications in order to adapt to biotic and abiotic stresses such as drought, nutrient deficiency, heavy metal and so on (de Dorlodot et al. 2007). All these heterogeneous events change during root development and some can result in the senescence or death of certain roots or the whole root system (Begara-Morales et al. 2013). Different from young, dividing and expanding meristematic zone in root tips, mature and senescent zone of rice root forms vascular tissues and disintegrated spongy cortex (aerenchyma). In this study, transverse sections showed that Cd treatment accelerated rice roots presenting mature and senescent symptoms such as promoted aerenchyma development and reduced parenchyma cells (Fig. 2), these mature and senescent symptoms can not only facilitate net O_2_ flux uptake but also decrease net Cd^2+^ flux uptake in rice roots (Fig. 3). All of these evidences indicate that Cd treatment can accelerate root mature and senescence of rice roots.

Oxygen is positive for the growth of rice, aerobic cultivation and other methods to increase the amount of rhizosphere oxygen can significantly promote the growth of rice (Zhu et al. 2015; Yang et al. 2017). In this study, aeration treatment promoted rice seedling growth, especially root growth, significantly (Fig. 1). Transverse sections showed that aeration increased cell expansion and reduced aerenchyma development (Fig. 2). Aeration not only partly recovered Cd-inhibited root growth but also inhibited formation of disintegrated spongy cortex. As a wetland plant, rice forms continuous and spongy aerenchyma tissue facilitating gas transport from and to the aerial parts of the seedling. However, the development of gas-impermeable aerenchyma seems likely to impair the ability of the root to absorb nutrients (Kirk 2003). It is a possible strategy for rice reducing Cd toxicity, because Cd-induced formation of aerenchyma seems likely to reduce Cd absorption in rice root. In addition, root aeration supplies sufficient oxygen for rice seedling growth, so it is not worth to impair nutrient absorption to develop gas-impermeable aerenchyma. Aerenchyma formation is through programmed cell death in the root cortex (Steffens et al. 2011), it is a broad consensus that aerenchyma formation in flooded roots is triggered by accumulation of ethylene, Ca^2+^ and reactive oxygen species (ROS) signaling (Drew et al. 2000; Evans 2004; Rajhi et al. 2011; Yamauchi et al. 2014).

### Aeration Enhances Cd Retention on Root Surface by Increasing Iron Plaque

For a long time, the formation of iron plaque is due to the radial oxygen loss (ROL) and oxidants in the rhizosphere, where ferrous is subsequently oxidized to ferric iron with the precipitation of iron oxide or hydroxide on the root surface (Chen et al. 1980; Taylor et al. 1984). Here, we newly reported that root aeration, an exogenous O_2_ supply, in hydroponic experiment also significantly increased iron plaque formation on rice root surface (Fig. 4). Aeration also increased malondialdehyde (MDA) content in the roots under aeration, even if aeration decreased the MDA content of rice shoots (Additional file 1: Figure S1). MDA is a product of membrane lipid peroxidation in plant tissues and indirectly reflects oxidation stress. It’s possible that aeration increase ROS and form iron plaques on the roots indirectly, but more direct evidences are still needed. More interesting, Cd treatment also increased iron plaque significantly in both presence and absence of aeration. All these evidences indicated that ROL is not the only reason for iron plaque formation on the surface of rice roots.

Iron plaque is considered can act as a filter for toxic metals, reacting and therefore immobilizing and preventing toxic metals uptake (Pi et al. 2010). Previous studies have shown that iron plaque can immobilize heavy metals such as Cd by adsorption and coprecipitation (Du et al. 2013). In this research, we also found a relative high concentration (about 900 μg/g DW) of Cd in the iron plaque of rice roots (Fig. 4c). Furthermore, aeration significantly increased Cd retention in iron plaque (Fig. 4c). In addition, results of NMT analysis showed that aeration increased net Cd^2+^ flux on the surface of rice roots (Fig. 3b, e). All of these results showed that aeration enhances Cd retention on root surface by increasing plaque. These results are consistent with previous study, which got similar result with aeration treatments on iron plaque formation (Wu et al. 2012).

Compared with a relative high concentration (about 900 μg/g DW) in iron plaque on the surface of rice root, Cd concentration in roots is slightly higher (Fig. 5b). Aeration increased Cd content in rice roots, but it decreased Cd content in the shoots (Fig. 5a). Similarly, Yang et al. (2009) reported that moderate soil drying and severe soil drying increased Cd content in roots while they reduced it in the straw. Following uptake by roots, Cd is transported to shoots via xylem and phloem, where exist a large amount of vascular bundles (Yoneyama et al. 2015). NMT analysis showed that aeration decreased net Cd^2+^ flux in the xylem of rice roots (Fig. 3h). All of these evidences indicate that aeration increased Cd retention in iron plaque and inhibited Cd translocation from the roots to the shoots by reducing xylem loading.

Previous researches showed that moderate or internal soil drying increased Cd accumulation in rice roots (Yang et al. 2009). Cd presented as free Cd^2+ ^in soil under aerobic condition was thought to be the reason for increased Cd content in rice (Du Laing et al. 2009). Here, our hydroponic experiment results also showed that aeration increased Cd accumulation in rice roots (Fig. 5b). It seems that besides increasing free Cd^2+^ in soil, O_2_ also increases Cd accumulation in rice roots through other ways. So, further studies are needed to discover the roles of O_2_ in regulating Cd accumulation in rice roots.

### Aeration Enhances Cd Retention in Root Cell Wall by Regulating Pectin Synthesis

Results of root cell fraction analysis showed that Cd in rice roots mainly accumulated in the cell wall and aeration increased Cd accumulation in the cell wall rather than cell organelle or soluble fraction (Fig. 6). There is a close positive correlation between pectin content and Cd accumulation in cell walls (Xiong et al. 2009b). Further researches by Paynel et al. (2009) have indicated that Cd induces an increase in PME activity and causes a decrease of pectin methylesterification degree, which determines negative charges and has a close negative correlation with heavy metal adsorption in the cell walls of plant roots (Eticha et al. 2005; Yang et al. 2008). As previous report (Xiong et al. 2009b), Cd treatment increased pectin, HC1 and HC2 content in rice roots (Fig. 7). Moreover, aeration also increased pectin content in rice in the absence of Cd, enzyme activity analysis showed that aeration increased PME activity and decreased pectin methylesterification degree in the cell wall of rice roots (Fig. 8). All of these results suggested aeration enhances Cd retention in root cell wall by regulating pectin synthesis and methylesterification. In addition, our previous report has showed that exogenous H_2_O_2_ not only increased pectin content but also increased PME activity and decreased pectin methylesterification degree in rice roots (Xiong et al. 2015). So that, further studies are need to investigate the effect of aeration on H_2_O_2_ accumulation in rice roots. In addition, aeration may increase pectin synthesis by delaying root mature and senescence, because pectin is mainly synthesized in young, dividing and expanding cell in elongation and meristematic zones of roots.

### Cd Stress and Aeration Increases Fe Accumulation in Iron Plaque while Decreases Fe Accumulation in Roots and Shoots of Rice Seedlings

It’s well-known that there is competitive interaction between Fe and Cd due to similar transport route within plants (Sharma et al. 2004). In resistance to Cd, plants tend to take up more Fe and act as a defence mechanism to prevent Cd from entering into root (Astolfi et al. 2014; Gong et al. 2015). Several studies showed that exogenous Fe supply resulted in decreased Cd uptake while increased Fe uptake in rice (Liu et al. 2008; Shao et al. 2008; Sebastian and Prasad 2015). According to the competitive interaction between Cd and Fe, Cd stress should decrease rather than increase Fe uptake in rice roots, but previous reports (Yoshihara et al. 2006; Meda et al. 2007; Gao et al. 2011), including our previous report (Yang et al. 2016), showed that Cd treatment significantly increased Fe content in root. Why did this “contradiction” happen?

Here, our results discovered the explanation for this “contradiction”. If fact, Cd treatment mainly increased Fe content in iron plaque on the surface of rice roots (Fig. 4b), but it indeed decreased Fe contents in both shoots and roots (after the DCB extraction of iron plaque) of rice seedlings (Fig. 5). This result indicates that it is necessary to consider and wash down iron plaque before determining real metal concentration in rice roots.

As to Fe content, aeration increased Fe content in roots but had no obvious effect on Fe content in shoots in rice seedlings without Cd treatment (Fig. 5c, d). These results indicate Fe was restricted in roots too. After absorbed by the roots, Fe can be transported to the vascular bundle by both apoplast and symplast system, and then transported to the shoots through the xylem and phloem (Yoneyama et al. 2015). In addition to the symplast pathway, the apoplast pathway is also an important transport route. And the cell wall is an important component of the apoplast. Due to the increase of pectin content in the cell wall caused by aeration (Figs. 7, 8), the binding ability of cell walls to Fe also increased. However, as shown in Additional file 1: Figure S2), aeration had different effect on Cu, Zn and Mn content in rice roots and shoots, indicating pectin binding is not the unique strategy for restricting Fe and Cd in the cell wall. In addition, the effects of aeration on Fe transporter expression remains unclear, more researches are still required to discover the detailed mechanism.

## Conclusions

Aeration, a method different from moderate or internal soil drying in increasing rhizosphere oxygen amount, alleviates Cd toxicity to rice seedlings by increasing net O_2_ and Cd^2+^ influxes on the surface of rice root. Then the increased net O_2_ influx delays Cd-accelerated mature and senescence of roots, increases iron plaque formation and Cd retention, promotes pectin synthesis and demesterification in the cell wall and decreases Cd translocation from roots to shoots.

## Additional file

Additional file 1: **Figure S1.** Effects of aeration or/and 50 μM CdCl_2_ treatments on malondialdehyde (MDA) content in shoots and roots of rice seedling. (a) MDA content in shoots; (b) MDA content in roots. The 3-week-old rice seedlings under hydroponic culture were aerated with air pump (30 min per hour) in the absence or presence of 50 μM CdCl_2_ for 14 d. The values are means ± SE (*n* = 3). Different letters on bar indicate significant differences at *P* < 0.05. **Figure S2.** Effects of aeration or/and 50 μM CdCl_2_ treatments on Cu, Zn and Mn content in shoots and roots of rice seedling. (a) Cu content in shoots; (b) Cu content in roots; (c) Zn content in shoots; (d) Zn content in roots; (e) Mn content in shoots; (f) Mn content in roots; The 3-week-old rice seedlings under hydroponic culture were aerated with air pump (30 min per hour) in the absence or presence of 50 μM CdCl_2_ for 14 d. The values are means ± SE (*n* = 3). Different letters on bar indicate significant differences at *P* < 0.05. (DOCX 2357 kb)

## Abbreviations

DCB: Dithionite–citrate–bicarbonate method
MD: Re-watered when soil water potential decreased to −20 kPa
NMT: On-invasive Micro-test Technology
PC: Phytochelatins
PMD: Pectin methylesterification degree
PME: Pectin methylesterase
SD: Re-watered when soil water potential decreased to −40 kPa
