# Supplementary material for: Aeration Increases Cadmium (Cd) Retention by Enhancing Iron Plaque Formation and Regulating Pectin Synthesis in the Roots of Rice (Oryza sativa) Seedlings
Source: Rice (N Y). 2019 May 2;12:28. doi: 10.1186/s12284-019-0291-0 (PMC6497704; doi:10.1186/s12284-019-0291-0)
Supplement: Supplementary file 1 — Figure S1. Effects of aeration or/and 50 μM CdCl2 treatments on malondialdehyde (MDA) content in shoots and roots of rice seedling. (a) MDA content in shoots; (b) MDA content in roots. The 3-week-old rice seedlings under hydroponic culture were aerated with air pump (30 min per hour) in the absence or presence of 50 μM CdCl2 for 14 d. The values are means ± SE (n = 3). Different letters on bar indicate significant differences at P < 0.05. Figure S2. Effects of aeration or/and 50 μM CdCl2 treatments on Cu, Zn and Mn content in shoots and roots of rice seedling. (a) Cu content in shoots; (b) Cu content in roots; (c) Zn content in shoots; (d) Zn content in roots; (e) Mn content in shoots; (f) Mn content in roots; The 3-week-old rice seedlings under hydroponic culture were aerated with air pump (30 min per hour) in the absence or presence of 50 μM CdCl2 for 14 d. The values are means ± SE (n = 3). Different letters on bar indicate significant differences at P < 0.05. (DOCX 2357 kb) [file 12284_2019_291_MOESM1_ESM.docx]

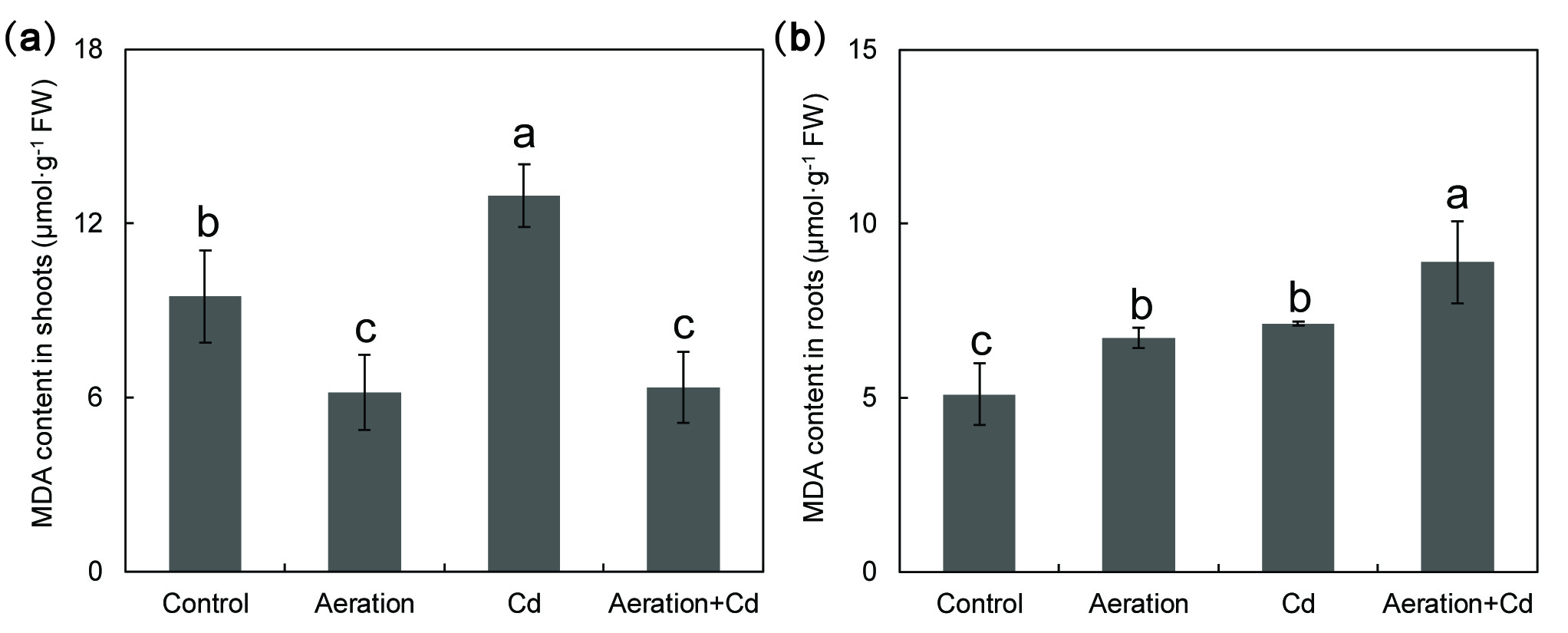


Fig. S1 Effects of aeration or/and 50 µM CdCl_2_ treatments on malondialdehyde (MDA) content in shoots and roots of rice seedling. (a) MDA content in shoots; (b) MDA content in roots. The 3-week-old rice seedlings under hydroponic culture were aerated with air pump (30 min per hour) in the absence or presence of 50 µM CdCl_2_ for 14 d. The values are means ± SE (n =3). Different letters on bar indicate significant differences at *P* < 0.05.


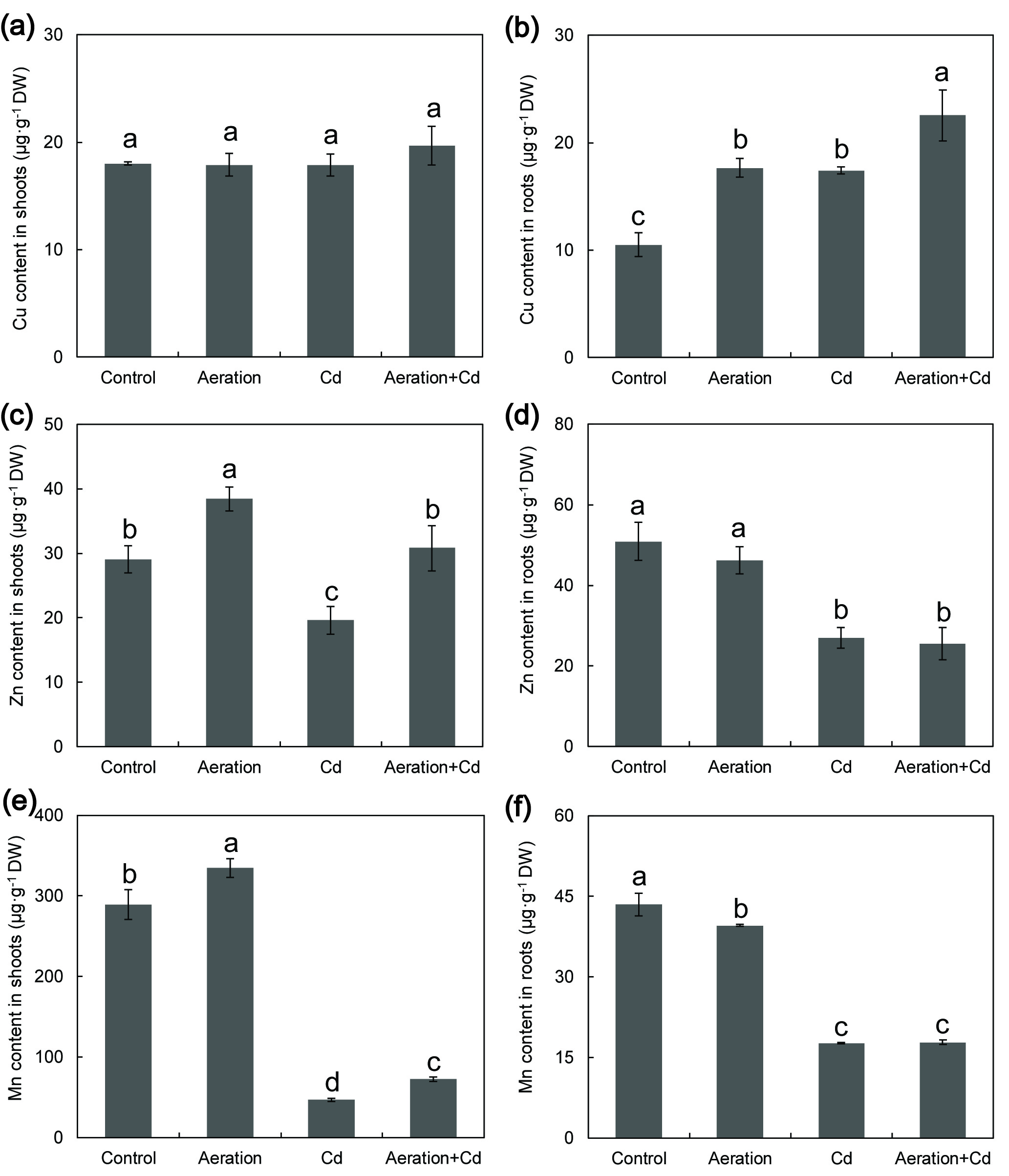


Fig. S2 Effects of aeration or/and 50 µM CdCl_2_ treatments on Cu, Zn and Mn content in shoots and roots of rice seedling. (a) Cu content in shoots; (b) Cu content in roots; (c) Zn content in shoots; (d) Zn content in roots; (e) Mn content in shoots; (f) Mn content in roots; The 3-week-old rice seedlings under hydroponic culture were aerated with air pump (30 min per hour) in the absence or presence of 50 µM CdCl_2_ for 14 d. The values are means ± SE (n =3). Different letters on bar indicate significant differences at *P* < 0.05.
